# Supplementary material for: Genetic architecture of band neutrophil fraction in Iceland
Source: Commun Biol. 2022 Jun 1;5:525. doi: 10.1038/s42003-022-03462-1 (PMC9160026; doi:10.1038/s42003-022-03462-1)
Supplement: Supplementary file 2 — Supplemental Information [file 42003_2022_3462_MOESM2_ESM.pdf]

Supplementary Information

Supplementary Table 1. Table showing general statistics of the Band neutrophil fraction phenotype used in the study, stratified by gender and two coding variants in LBR (p.Tyr430Cys and p.Arg76Ter). N: Number of individuals, Mean: The band neutrophil fraction mean value, SD: Standard deviation, Range: the range for band neutrophil fraction values, N measurements: number of measurements for band neutrophil fraction, mean N meas.: Mean number of measurements for band neutrophil fraction, AOM: Age of measurement.

	**Total phenotype**			**rs138769892**			**p.Arg76Ter**		
	**All**	**Men**	**Women**	**All**	**Men**	**Women**	**All**	**Men**	**Women**
**N**	88,101	42,370	45,731	309	138	171	4	2	2
**Mean (%)**	8.4	8.4	8.4	13.7	13.4	14	28.5	25.5	31.5
**25% Q (%)**	2	2	2	5	6	4.6	22.6	24.8	25
**Median (%)**	5.5	5.5	5.5	10	10.4	10	25.5	25.5	31.5
**75% Q (%)**	11.6	11.6	11.6	19	18.9	19.3	31.4	26.3	38
**SD**	9	9	9	12.1	11.2	12.8	11.2	2.1	18.4
**Range (%)**	0;85	0;84	0;85	0;75	0;56	0;75	18.5;44.5	24;27	18.5;44.5
**N meas**	258,312	126,698	131,614	956	477	479	16	4	12
**Mean N meas**	2.9	3	2.9	3.1	3.5	2.8	4	2	6
**AOM 25% Q**	13	7	18	43	64	75	56	60	56
**AOM median**	52	52	52	45	65	77	56	69	56
**AOM 75% Q**	72	71	72	39	61	72	63	69	57

Supplementary Table 2. Heritability (additive model) estimated for band neutrophil proportion using parent-offspring regression and sibling regression, SE: standard error

Phenotype	Heritability, parent-offspring regression (SE)	Heritability, full sibling regression (SE)
Band neutrophil fraction	0.10 [95% CI: 0.08-0.12]	0.14 [95% CI: 0.12-0.17]

Supplementary Table 3. Genetic correlation between band neutrophil fraction from Iceland and eight quantitative blood traits from the UK using LD score regression.

	Neutrophil bands		
**Secondary trait**	r_g	**SE**	***P*-value**
Basophils	0.1322	0.0824	0.1088
Eosinophils	0.0377	0.0673	0.5753
Lymphocytes	-0.0628	0.0612	0.3054
Monocytes	-0.0591	0.0557	0.2887
Neutrophils	0.142	0.0645	0.0278
Platelets	0.0112	0.0432	0.7947
Red blood cells	0.0609	0.0585	0.2978
White blood cells	0.0913	0.0636	0.1509

r_g = genetic correlation, SE= standard error

Supplementary Table 4. Protein quantitative trait loci (pQTL) signals conferred by the band neutrophil fraction associated variants or by variants highly correlated with the band neutrophil fraction associated variants. Results from measurements of 4,730 plasma proteins measured in 35,559 Icelanders using SOMAscan. Effect is shown for the minor allele in standard deviations. Loci: the loci or genomic region the variant belongs to, probe target: protein target the probe targets, target gene: the gene encoding the protein target, mLog10(pval): logarithm base 10 of the P-value.

**Variant**	**Loci**	**pQTL marker (Hg38)**	**R2**	**Probe Target**	**Target gene**	**Gene chromosome**	**Cis or trans**	**Effect (SD)**	**mLog10(pval)**
**rs724781 (chr1:153363542)**	*S100A9*	rs3014874 (chr1:153365467)	0.87	Lipocalin 2	*LCN2*	chr9	trans	0.06	9.2
**rs724781 (chr1:153363542)**	*S100A9*	rs3014874 (chr1:153365467)	0.87	Protein S100-A12	*S100A12*	chr1	cis	-0.22	114.81
**rs12613605 (chr2:43131771)**	*2p21*	rs13390874 (chr2:43126091)	0.96	Eosinophil cationic protein	*RNASE3*	chr14	trans	0.06	9.28
**rs12613605 (chr2:43131771)**	*2p21*	rs13390874 (chr2:43126091)	0.96	Resistin	*RETN*	chr19	trans	0.09	18.84
**rs12613605 (chr2:43131771)**	*2p21*	chr2:43132699	0.93	Peptidoglycan recognition protein 1	*PGLYRP1*	chr19	trans	0.06	11.13
**rs7846314 (chr8:60738272)**	*CHD7*	chr8:60733793	0.99	Carcinoembryonic antigen-related cell adhesion molecule 8	*CEACAM8*	chr19	trans	0.21	84.46
**rs7846314 (chr8:60738272)**	*CHD7*	chr8:60733793	0.99	Lactotransferrin	*LTF*	chr3	trans	0.06	9.26
**rs7846314 (chr8:60738272)**	*CHD7*	rs13256023 (chr8:60735992)	0.99	Pappalysin-1	*PAPPA*	chr9	trans	-0.13	33.61
**rs7846314 (chr8:60738272)**	*CHD7*	rs13256023 (chr8:60735992)	0.99	EMBP	*PRG2*	chr11	trans	-0.06	9.6
**rs7846314 (chr8:60738272)**	*CHD7*	rs13256023 (chr8:60735992)	0.99	Proteoglycan 3	*PRG3*	chr11	trans	-0.12	31.29
**rs7846314 (chr8:60738272)**	*CHD7*	rs7846314 (chr8:60738272)	1.00	Lipocalin 2	*LCN2*	chr9	trans	0.08	14.85
**rs36084354 (chr19:1079960)**	*HMHA1*	rs36084354 (chr19:1079960)	1.00	sL-Selectin	*SELL*	chr1	trans	0.07	9.19

Supplementary Table 5. Conditional analysis of the variants in the Icelandic dataset representing associations with band neutrophil fraction, where each variant is tested with the other variants on the locus associated with the trait as covariates. All but two LBR variants r2<0.02 (rs41268715[C] and rs80028106[T] r2=0.17). Effect is shown for the minor allele in standard deviations. HGVS: Human genome variation society nomenclature; LD-class: total number of variants correlating with R2 > 0.8 to the variant (stratified by functional impact class, where HIGH impact variants include stop-gained, frameshift, splice acceptor or donor; MODerate impact variants include missense, splice-region variants and in-frame indels; LOW impact variants include upstream and downstream variants; and LOWEST impact variants include intron and intergenic variants); Amin: Minor allele; Amaj: Major allele

**Rs_name**	**Position(Hg38)**	**Gene or loci**	**HGVS**	**LD class**	**Amin/ Amax**	**P unadjusted**	**P adjusted**	**Effect unadjusted**	**Effect adjusted**
rs724781	chr1:153363542	S100A9	.	7 (0/0/5/2)	G/C	6.6E-12	.	-0.05	.
rs2245425	chr1:179889309	TOR1AIP1	NM_001267578.1:c.554-1G>A	74 (1/2/24/47)	G/A	6.9E-08	.	0.03	.
rs41268715	chr1:225333399	DNAH14	NP_001364.1:p.Glu3232Gln	39 (0/2/0/37)	C/G	3.2E-36	9.6E-11	-0.14	-0.08
rs17522489	chr1:225346229	DNAH14	NP_001364.1:p.Gln3556Arg	26 (0/2/0/25)	G/A	5.5E-09	8.7E-11	0.04	0.05
rs14205	chr1:225401942	LBR	NM_194442.2:c.*1361T>C	18 (0/1/4/13)	G/A	3.1E-41	2.5E-43	0.12	0.13
rs80028106	chr1:225402647	LBR	NM_194442.2:c.*656G>A	4 (0/0/2/2)	T/C	8.6E-30	5.9E-11	-0.24	-0.15
rs138769892	chr1:225410316	LBR	NP_919424.1:p.Tyr430Cys	33 (0/2/5/26)	C/T	9.1E-25	1.1E-26	0.51	0.53
rs12613605	chr2:43131771	2p21	.	53 (0/0/0/53)	T/G	3.2E-10	.	0.04	.
rs2036844	chr5:126776461	LMNB1	.	21 (0/0/1/20)	C/A	2.3E-15	.	0.05	.
rs2561758	chr5:173778279	5q35.2	.	7 (0/0/0/7)	A/G	6.8E-13	.	0.05	.
rs757770077	chr7:65987227	GUSB	.	576 (0/0/50/526)	*	2.2E-09	.	-0.06	.
rs7846314	chr8:60738272	CHD7	.	4 (0/0/1/3)	T/A	3.6E-16	.	0.06	.
rs36084354	chr19:1079960	HMHA1	NP_001245257.1:p.Met531Ile	3 (0/1/1/1)	A/G	3.1E-14	.	0.07	.

*CTTTCTTTCTTTCTTTCTTTTTTTTTT/!

Supplementary Table 6: Linkage disequilibrium between variants at the LBR locus displayed as correlation (r^2)

Variant (rs number)	Position (Hg38)	LD (r^2)				
		**rs41268715**	**rs17522489**	**rs14205**	**rs80028106**	**rs138769892**
rs41268715	chr:1225333399	1.00	-	-	-	-
rs17522489	chr1:225346229	0.01906	1.00	-	-	-
rs14205	chr1:225401942	0.010133	0.028841	1.00	-	-
rs80028106	chr1:225402647	0.168816	0.004808	0.002673	1.00	-
rs138769892	chr1:225410316	0.000246	0.000886	0.000472	0.000065	1.00

Supplementary Table 7: Pelger Huët Pedigree in Iceland.

		Band neutrophil level		
Individual	Pelger-Huët	Standardised (SD)	Percentile	Arg76Ter status
I.1	Unknown	-	-	Unknown
I.2	Unknown	-	-	Unknown
II.1	Unknown	-	-	Obligate
II.2	Unknown	-	-	Unknown
II.3	Unknown	-	-	Unknown
II.4	Unknown	-	-	Unknown
II.5	Unknown	-	-	Unknown
II.6	Unknown	-	-	Obligate
III.1	Unknown	-	-	Unknown
III.2	Unknown	-	-	Obligate
III.3	Unknown	-	-	Unknown
III.4	Unknown	-	-	Unknown
III.5	Unknown	-	-	Obligate
III.6	Unknown	-	-	Unknown
IV.1	Yes	-	-	Obligate
IV.2	Unknown	-	-	Unknown
IV.3	Unknown	-	-	Unknown
IV.4	Unknown	-	-	Obligate
V.1	Yes	-	-	Obligate
V.2	Unknown	1.10	86th	Unknown
V.3	Yes	-	-	Obligate
V.4	Unknown	-	-	Unknown
V.5	Yes	-	-	Obligate
V.6	Unknown	-	-	Unknown
V.7	Unknown	-	-	Unknown
V.8	Yes	-	-	Unknown
V.9	No	-	-	Unknown
V.10	Unknown	-	-	Unknown
V.11	Yes	-	-	Unknown
V.12	Unknown	-	-	Unknown
V.13	Yes	-	-	Obligate
V.14	No	-	-	Non-carrier
VI.1	Unknown	-	-	Unknown
VI.2	Yes	2.40	99th	Carrier
VI.3	Yes	2.20	98th	Unknown
VI.4	No	-	-	Non-carrier
VI.5	Yes	1.30	91st	Carrier
VI.6	No	-	-	Non-carrier
VI.7	Unknown	-	-	Unknown
VI.8	Yes	-	-	Carrier
VI.9	No	2.10	98th	Unknown
VI.10	No	-	-	Non-carrier
VI.11	No	-	-	Non-carrier
VI.12	No	-	-	Non-carrier
VI.13	No	-	-	Non-carrier
VI.14	No	0.52	70th	Unknown
VI.15	No	-	-	Non-carrier
VI.16	No	1.30	91st	Unknown
VI.17	No	0.33	63rd	Unknown
VI.18	Yes	-	-	Unknown
VI.19	Yes	-	-	Unknown
VI.20	Yes	-	-	Carrier
VI.21	Yes	1.00	85th	Carrier
VII.1	Unknown	-	-	Carrier
VII.2	Unknown	-	-	Unknown
VII.3	Unknown	1.60	94th	Carrier
VII.4	Unknown	-	-	Carrier
VIII.1	Unknown	2.00	97th	Unknown

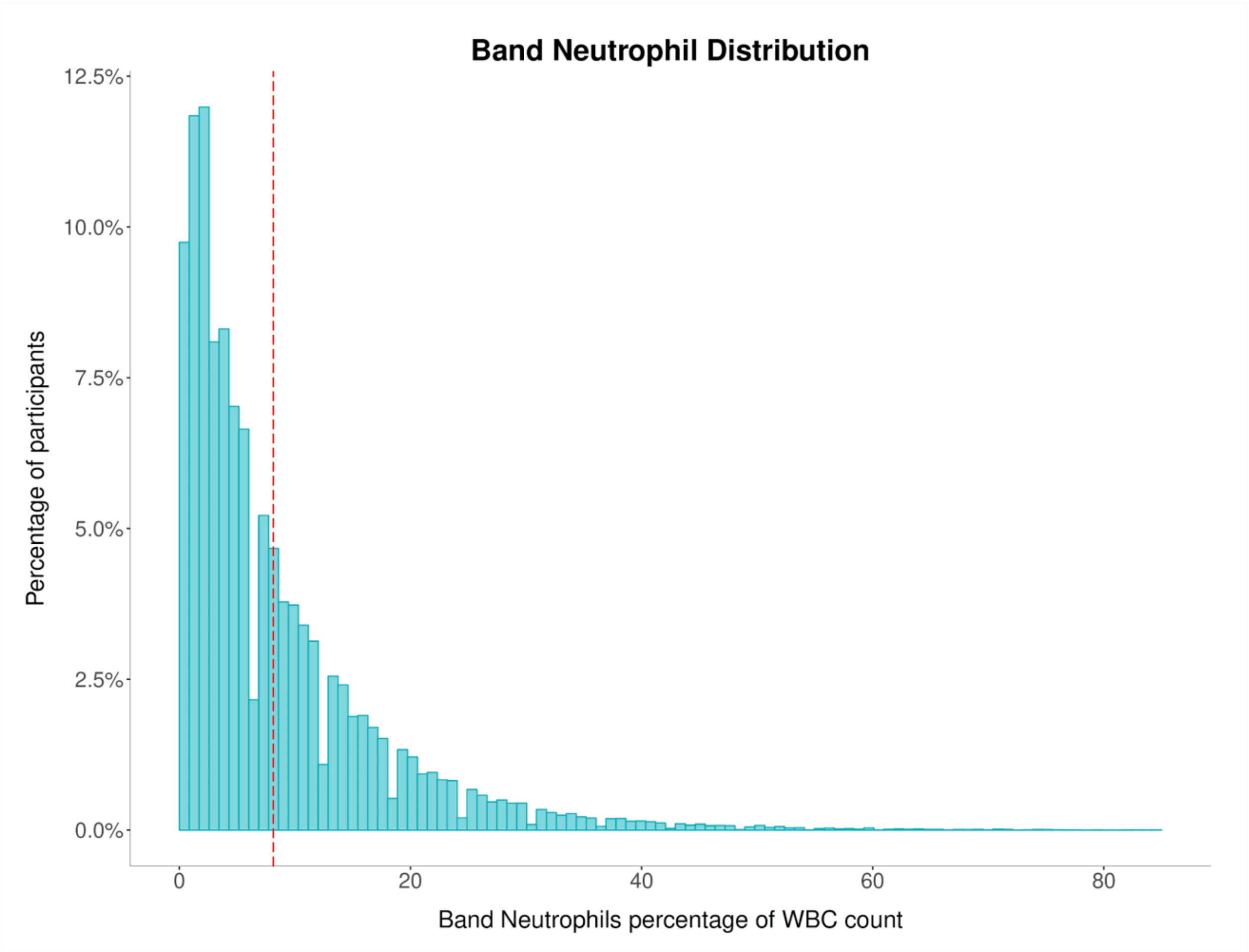

Supplementary Figure 1. Distribution of mean band neutrophil fraction per individual (N = 88,101) in the Icelandic population. Y-axis represents the fraction of individuals with band neutrophil fraction assessments. X-axis represents the band neutrophil fraction. Red dashed line shows the median band neutrophil fraction in the population. Band neutrophil fraction is defined as the percentage of WBC count made up of band neutrophils.

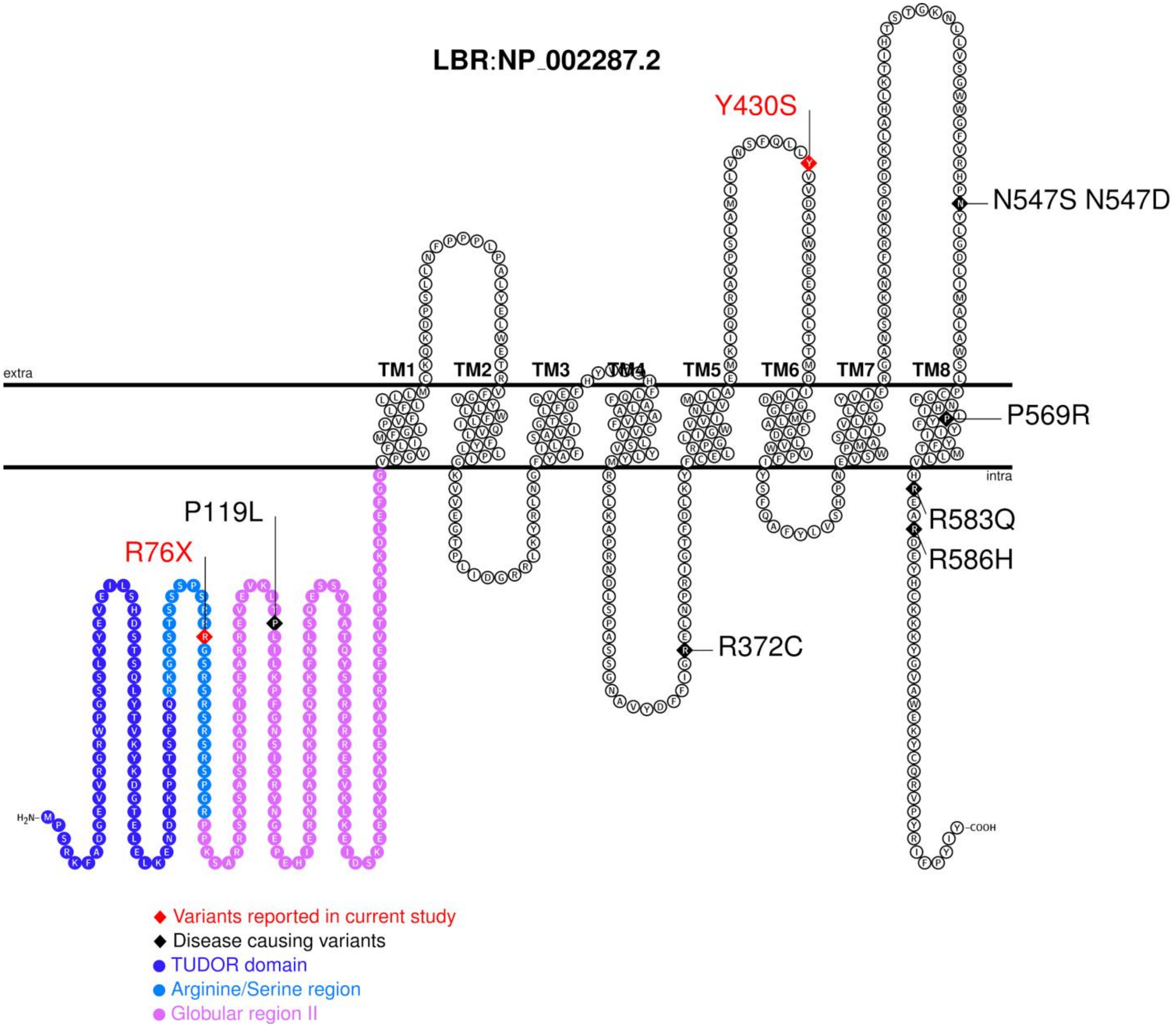
LBR:NP_002287.2
Y430S
N547S N547D
P569R
R583Q
R586H
R372C
P119L
R76X
extra
intra
TM1
TM2
TM3
TM4
TM5
TM6
TM7
TM8
H2N-
-COOH
Variants reported in current study
Disease causing variants
TUDOR domain
Arginine/Serine region
Globular region II

Supplementary Figure 2. Schematic figure depicting the structure of the lamin B receptor (LBR:NP_002287.2) anchored to the inner nuclear membrane. LBR consists of an N-terminal nucleoplasmic domain interacting with lamin B and the heterochromatin (TUDOR domain: aa. 1-61, arginine/serine region: aa. 63-89, and second globular region: aa. 90-211), followed by eight transmembrane (TM) segments with sterol reductase activity, and a C-terminal nucleoplasmic domain[1]. Mutations reported in cases of Pelger-Huët and/or Greenberg dysplasia are shown on the figure and implicated by their amino acid substitution. The rare missense variant Y430S (MAF: 0.33%), associated with band neutrophil fraction in the Icelandic population, has not been reported in cases of Pelger-Huët anomaly nor Greenberg dysplasia. The stop-gained variant R76X is the causal mutation for a familial Pelger-Huët anomaly previously reported in articles published in 1963 and 1977 by Jensson & Arnason[2] and Jensson et. al.[2,3], respectively.

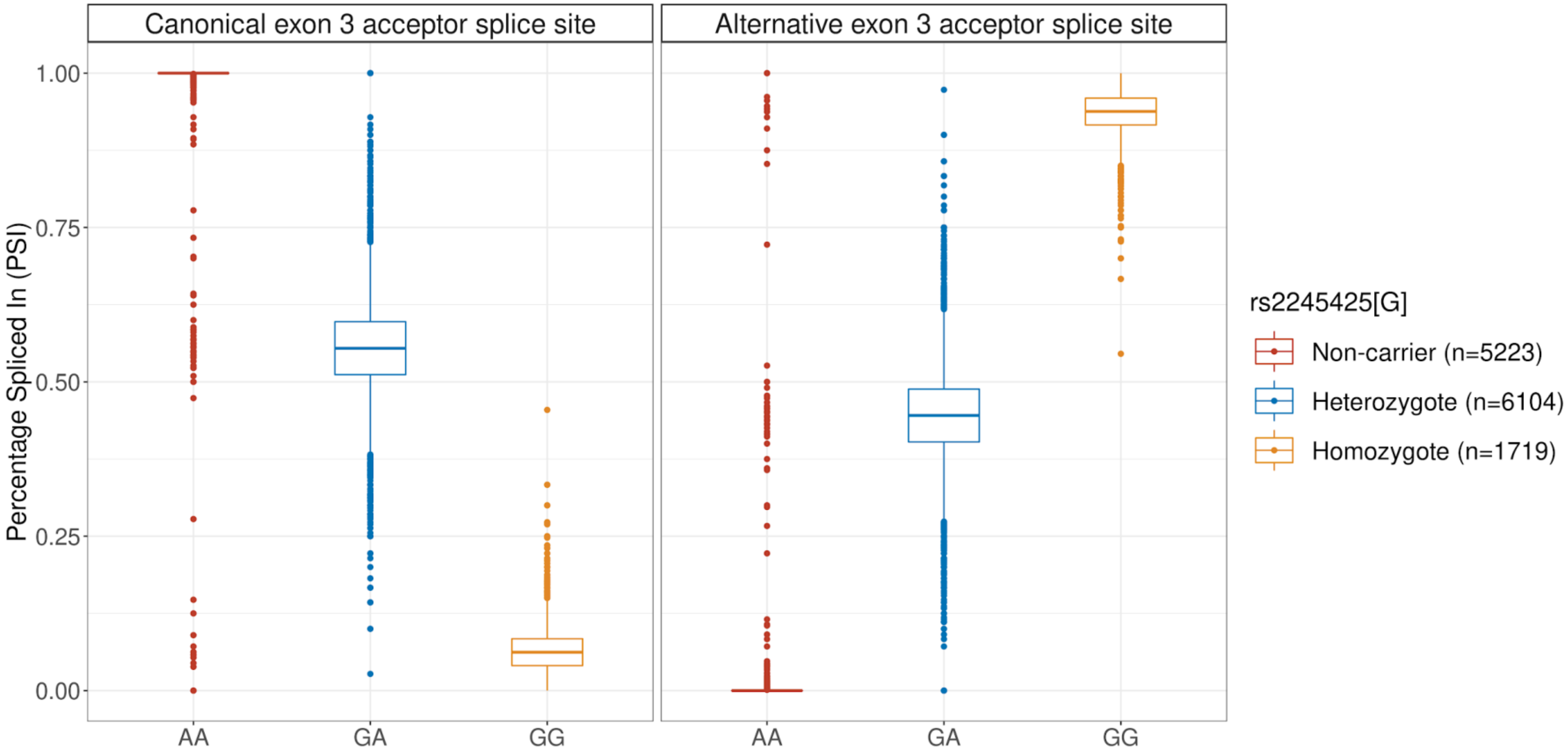

Supplementary Figure 3. Quantification of proportional usage (percentage spliced in values) of the two alternative acceptor splice sites of exon 3 in *TOR1AIP1* starting from exon 2, stratified by rs2245425[G] genotype. The usage of the alternative acceptor splice site of exon 3 increases significantly (effect = 1.28, p<1x10-300) while the canonical acceptor splice site of exon 3 decreases accordingly (effect = -1.28, p<1x10-300). After removing 82 samples due to lack of expression, 13,046 samples were used for the analysis (Methods). The hinges of the boxes represent the quartiles of the distribution, and whiskers represent the highest and lowest point no further than 1.5 times the interquartile range from the upper and lower hinges, respectively.

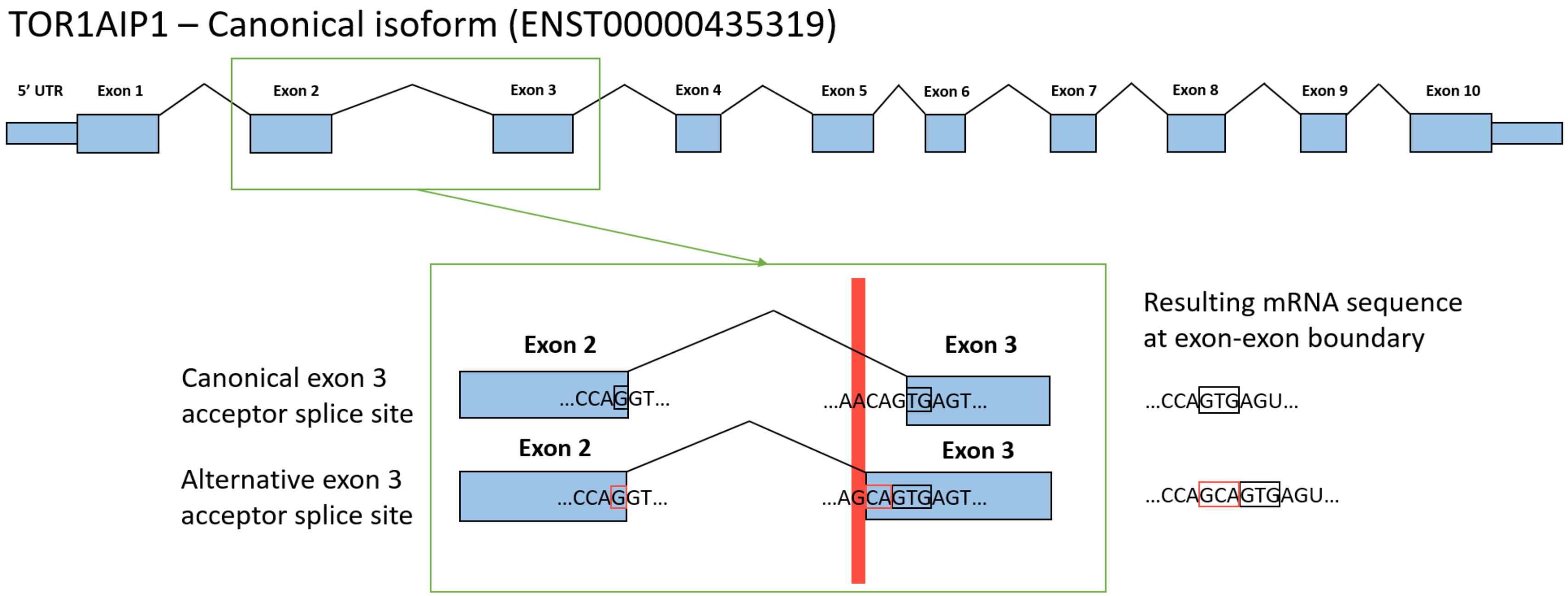

Supplementary Figure 4. A schematic illustration of the effect of rs2245425[G] in *TOR1AIP1* on RNA-splicing. Exons are enumerated according to ENST00000435319, the canonical isoform in whole blood (the isoform with the highest median expression in whole blood). The variant is located four base pairs upstream of exon 3, inducing a splice site three base pairs upstream, adding Arginine (GCA) to the resulting mRNA isoform. Note that the alternatively spliced isoform is annotated in ENSEMBL (ENST00000528443).

Supplementary references

1. Nikolakaki, E., Mylonis, I. & Giannakouros, T. Lamin B Receptor: Interplay between Structure, Function and Localization. *Cells* **6**, (2017).
2. Jensson, O. & Arnason, K. [AN ICELANDIC PELGER ANOMALY FAMILY]. *Laeknabladid* **47**, 97–101 (1963).
3. Jensson, O., Arnason, K., Jóhannesson, G. M. & Ulfarsson, J. Studies on the Pelger anomaly in Iceland. *Acta Med. Scand.* **201**, 183–185 (1977).
