# Supplementary material for: Genetic architecture of band neutrophil fraction in Iceland
Source: Commun Biol. 2022 Jun 1;5:525. doi: 10.1038/s42003-022-03462-1 (PMC9160026; doi:10.1038/s42003-022-03462-1)
Supplement: Supplementary file 5 — Description of Additional Supplementary Files [file 42003_2022_3462_MOESM5_ESM.pdf]

Description of Additional Supplementary Files

File name: Supplementary Data 1
Description: Expression quantitative trait loci (eQTL) signals conferred by the band neutrophil fraction associated variants or by variants highly correlated with the band neutrophil fraction associated variants. Results come from foreign databases (Supplementary Data 2) and RNA sequencing of Icelanders. Loci: the loci or genomic region the variant belongs to, R2: the LD correlation between band neutrophil fraction associated variant and eQTL marker, gene transcript: the transcript that is analysed, effect type: where the effect type is beta the effect is shown in standard deviation.

File name: Supplementary Data 2
Description: Sources for expression quantitative trait loci (eQTL) data shown in Supplementary Data 1.
